# Novel quantitative pigmentation phenotyping enhances genetic association, epistasis, and prediction of human eye colour

**DOI:** 10.1038/srep43359

**Published:** 2017-02-27

**Authors:** Andreas Wollstein, Susan Walsh, Fan Liu, Usha Chakravarthy, Mati Rahu, Johan H. Seland, Gisèle Soubrane, Laura Tomazzoli, Fotis Topouzis, Johannes R. Vingerling, Jesus Vioque, Stefan Böhringer, Astrid E. Fletcher, Manfred Kayser

**Affiliations:** 1 Department of Genetic Identification, Erasmus MC University Medical Center Rotterdam, Rotterdam, The Netherlands; 2 Department of Medical Statistics and Bioinformatics, Leiden University Medical Center, Leiden, The Netherlands; 3 Section of Evolutionary Biology, Department of Biology II, University of Munich LMU, Planegg-Martinsried, Germany; 4 Department of Biology, Indiana University-Purdue University Indianapolis, Indianapolis, IN, USA; 5 Key Laboratory of Genomic and Precision Medicine, Beijing Institute of Genomics, Chinese Academy of Sciences, Beijing, China; 6 Centre for Vision and Vascular Science, The Queen’s University Belfast, Belfast, United Kingdom; 7 Department of Epidemiology and Biostatistics, National Institute for Health Development, Tallinn, Estonia; 8 Department of Ophthalmology, University of Bergen, School of Medicine, Bergen, Norway; 9 Clinique Ophthalmologique, Universitaire De Creteil, Paris, France; 10 Clinica Oculistica, Universita degli studi di Verona, Italy; 11 Department of Ophthalmology, Aristotle University of Thessaloniki, School of Medicine, Thessaloniki, Greece; 12 Department of Ophthalmology, Erasmus MC University Medical Centre Rotterdam, Rotterdam, The Netherlands; 13 Dpto. Salud Publica Universidad Miguel Hernandez, Alicante, El Centro de Investigacion Biomedica en Red de Epidemiologıa y Salud Publica (CIBERESP), Elche, Spain; 14 Faculty of Epidemiology & Population Health, London School of Hygiene & Tropical Medicine, London, United Kingdom

## Abstract

Success of genetic association and the prediction of phenotypic traits from DNA are known to depend on the accuracy of phenotype characterization, amongst other parameters. To overcome limitations in the characterization of human iris pigmentation, we introduce a fully automated approach that specifies the areal proportions proposed to represent differing pigmentation types, such as pheomelanin, eumelanin, and non-pigmented areas within the iris. We demonstrate the utility of this approach using high-resolution digital eye imagery and genotype data from 12 selected SNPs from over 3000 European samples of seven populations that are part of the EUREYE study. In comparison to previous quantification approaches, (1) we achieved an overall improvement in eye colour phenotyping, which provides a better separation of manually defined eye colour categories. (2) Single nucleotide polymorphisms (SNPs) known to be involved in human eye colour variation showed stronger associations with our approach. (3) We found new and confirmed previously noted SNP-SNP interactions. (4) We increased SNP-based prediction accuracy of quantitative eye colour. Our findings exemplify that precise quantification using the perceived biological basis of pigmentation leads to enhanced genetic association and prediction of eye colour. We expect our approach to deliver new pigmentation genes when applied to genome-wide association testing.

Human eye colour is determined by the type, amount, and distribution of two forms of pigment produced in the melanocytes of the iris, eumelanin and pheomelanin. Eumelanin is a highly compact pigment, packed in ovoid eumelanosomes^1^, which absorbs nearly the full light spectrum and is perceived as dark-brown to black colour. Pheomelanin in contrast, is a more sparse pigment that reflects in contrast to eumelanosomes a much broader light spectrum and is perceived as yellow to red colour^1^,^2^. With the complete absence of both pigments, the light is reflected by the stroma of the iris, and the eye colour is perceived as grey to blue through Tyndall scattering^3^. As with many traits, the nature of human eye colour variation is continuous, spanning from the lightest shades of grey or blue to the darkest shades of brown or black^4^. Dark eye colour reflects the ancestral state in humans linked to their commonly believed origin in Africa, while light eye colour is assumed to be derived; shaped by positive selection perhaps due to sexual selection during European history^5^.

Several gene-mapping studies on eye colour were previously conducted by using manually defined phenotype categories^6^,^7^,^8^,^9^,^10^,^11^,^12^,^13^,^14^, inevitably oversimplifying the continuous nature of human eye colour variation. Although this incomplete use of the underlying basics of eye colour variation reduces the thoroughness of such studies, several eye colour genes were previously identified with this simplified phenotyping approach^5^,^6^,^7^,^8^,^9^,^10^,^11^,^12^,^13^. Moreover, it provides an accurate prediction from DNA with reasonably high accuracies for at least the extreme categories of blue and brown demonstrated via the IrisPlex system^15^, a system consisting of only six single nucleotide polymorphisms (SNPs) from six genes. Consequently, eye colour was one of the first externally visible characteristic for which the concept of Forensic DNA Phenotyping (FDP)^16^,^17^ was put into practice^15^, later followed by hair colour^18^ and most recently by skin colour^19^. However, as described elsewhere^20^ there is a desire to move pigmentation colour prediction from the currently applied categorical level to the continuous level. As prerequisite, this requires an understanding of the genes that determine eye colour in its fully continuous spectrum as well as methodology that allows the capture of continuous eye colour as accurately and completely as possible.

The first quantitative approach to measure eye colour was proposed by Frudakis^21^, who introduced two quantities representing the iris colour properties, i.e. the iris colour score, and the melanin index as derived from average luminosity (L) and colour reflectance values (C) from selected boxes in digital eye-imagery. The melanin index can be directly related to the amount of melanin that is known to decrease to extremely low levels (even complete absence) in blue eyes^22^,^23^. In the hue (*H*) and saturation (*S*) measurements introduced to eye colour quantification by Liu *et al*.^24^, *H* defines the colour itself, which can be related to the type of melanin having more red or yellow components. The *S* value describes the richness of a certain colour (defined in *H*) that is supposed to correlate with the amount of eu-or pheo-melanin. The *V* value is usually discarded, as it is supposed to rather represent the brightness due to different lighting conditions^24^,^25^. Digital quantification of eye images of thousands of Europeans using the H-S colour space and its use in a genome-wide association study allowed the identification of three new eye colour genes, not previously identified when using categorical eye colour^24^. This study clearly demonstrated the increase of power to find new genes when moving pigmentation phenotyping from the classical categorical approach to a quantitative approach. Beleza *et al*.^26^ averaged and normalized *B*-*G* values from the *RGB*-space (red, green, blue, value) and proposed a T-index quantity to describe the amount of melanin per iris. Recently, the CIE-L * a * b* values have been used in place of *H, S* values^27^ to quantify iris colour, where the *L* value describes the lightness, the a* the red/green, and b* the yellow/blue component, of the colour respectively. However, taking a quantity that is averaged over the iris as previous methods^21^,^23^,^24^,^27^ have done may obscure the different mixture proportions of pigments. An alternative has been proposed by Anderson *et al*.^28^, which includes a clustering of the segmented iris into blue and brown pixels deriving a ratio score (PIE score). Different types of pigmentation (i.e. eumelanin or pheomelanin), however, are not distinguished with this approach.

Here, we introduce an improvement of quantitative pigmentation phenotyping based on an automated segmentation of the iris followed by a measurement of the digital equivalents of eumelanin, pheomelanin, and total absence of any pigment in the iris. This clustering is based on manually predefined (assumed) image segments that depict eumelanin, pheomelanin and nonpigmented areas. We exemplified the advantage of this approach by an empirical analysis of high-resolution eye images from over 3000 individuals sampled from seven European countries (EUREYE study). By using genotypes of 12 SNPs previously involved in human eye colour variation that we generated in the same individuals, we demonstrate the impact of this novel pigmentation phenotyping approach on genetic association, epistasis, and prediction.

## Results and Discussion

### Detection and segmentation of the iris in digital imagery

**Figure 1:**
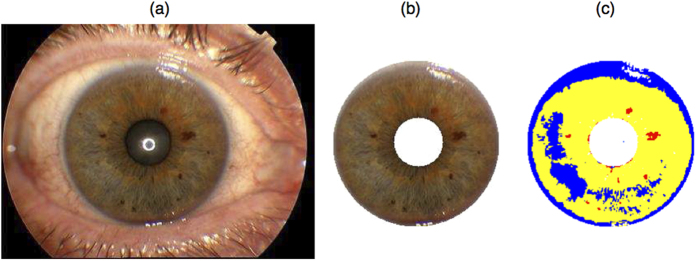
Example of fully automated iris segmentation and eye colour quantification using our new approach. Panel (a) shows the iris picture taken by the Topcon camera system used under normalized conditions. Panel (b) depicts the iris as automatically extracted with our iris segmentation approach. Panel (c) exemplifies the assignment of each pixel of the iris image into one of three types of clusters: non-pigmented areas (blue), pheomelanin (yellow), and eumelanin (red) with our new approach.

Prior to colour assessment, the iris needed to be segmented from the pupil and sclera. Several approaches have been proposed previously for the segmentation of the iris in digital eye imagery^28^,^29^,^30^,^31^,^32^. The utility of a certain approach depends strongly on the properties of the given image data. In the majority of imagery available to us, (i) the pupil was always centred in the middle (Fig. 1a), (ii) the iris was fully visible (those images where it was not were excluded from the analyses), and iii) eye lashes rarely overlapped with the iris. Because of these features, we followed a previously proposed^30^ two-step procedure, which we implemented in Matlab (R2007a). First, we used a Canny filter^33^ to distinguish the rim confining the iris and then applied the Hough transformation^34^ to detect the iris circle. To reduce the number of multiple solutions, we constrained the results of the Hough transformation on those circles only that were centred in the middle of the image. As a result, we were able to maintain a high ratio of correctly segmented irides (>90%). Falsely segmented irides, for example when the pupil was extremely dilated, or eyes were closed by chance, were curated manually. It may be that in other types of image data taken under less normalized conditions (i.e. DSR camera systems with macro lenses), other approaches^28^ might be more sensitive to apply for iris segmentation.

### Quantification of iris colour from digital imagery

The developed method assesses each pixel according to its digital classification of pheomelanin, eumelanin, and non-pigmentation, using a machine learning approach (Supplementary Figure S1). The colour information of each image pixel was available as a red-green-blue (RGB) triplet, which we first transformed into a hue-saturation-value (HSV) triplet^35^. We then used a support vector machine classifier^36^ with a quadratic kernel to assign each pixel within the HSV space to one of the manually defined classifications representing the total absence of pigment (non-pigmentation), pheomelanin, and eumelanin. To define the distribution of the respective classes for the training of the support vector machine (prior to the sample phenotyping procedure), we used a set of 10 randomly chosen images of different eye colours to manually label areas that most obviously contain the two types of pigments and their absence (Supplementary Figure S1a). The distributions of these three types were well separated in at least one dimension from the selected training images (Supplementary Figure S1b), which implies that the assignment of a pixel from the iris into one of the three types of pigment outcomes can be achieved with good precision. Note that the clustering outcomes were robust against the choice of the kernel and choice of colour model (RGB or HSV, data not shown).

We finally considered the proportion of the clustered pixels relative to the segmented iris as our quantitative eye colour phenotype, reflecting the equivalent amounts of non-pigmented, pheomelanin, and eumelanin areas per iris (see Fig. 1c), the sum of which equals to one.

### Comparing the new eye colour quantification method with previous methods

One important motivation behind quantitative measurements is to capture the information about complex phenotypes with a small number of variables. We use a correlation analysis to reveal how well different eye colour quantifications maintain the information about our estimated abundance of different types of melanin. We calculated quantitative iris colour phenotypes using five previously proposed methods: (i) the mean *H, S* values from the HSV space^24^ (ii) the mean luminosity value and colour score^21^, (iii) the components of the L * a * b* space^27^, (iv) the PIE score^28^ and (v) the T-index^26^.

Table 1 provides information about the relationship between the considered eye colour quantifiers. The amount of non-pigmentation we find mostly positively correlated with b* (r = 0.93, *P* < 1e–300), followed by *S* (−0.74, *P* < 1e–300). The more melanin is distributed on the iris (fewer non-pigmentation) the lower will be the b* indicating the yellow components contained in the melanin and the higher is the a* value for the red components of the melanin. Therefore we find the amount of pheomelanin was most strongly correlated with a* (r = 0.76, *P* < 1e–300), followed by b* (r = −0.68, *P* < 1e–300). The amount of eumelanin estimated with our approach was most strongly positively correlated with *L*^21^, (r = 0.71, P < 1e–300), followed by *S (r* = 0.69, *P* < 1e–300). The colour score *C*, which summarizes the components a* and b*^21^, shows a weaker correlation with eumelanin (*r* = −0.52, *P* = 1.1e–286) and much weaker positive correlation with pheomelanin (*r* = 0.14, *P* = 3.6e–14), which indicates a reduced utility in predicting the respective phenotypes from colour components, which is discussed in the next paragraph.

Finally, the amount of pheomelanin estimated with our approach showed only weak correlation with the estimated amount of eumelanin (*r* = −0.06, *P* = 0.001), confirming our expectation that these two measurements represent different biological phenotypes.

### Comparing quantitative eye phenotypes with categorical eye phenotypes

**Figure 2:**
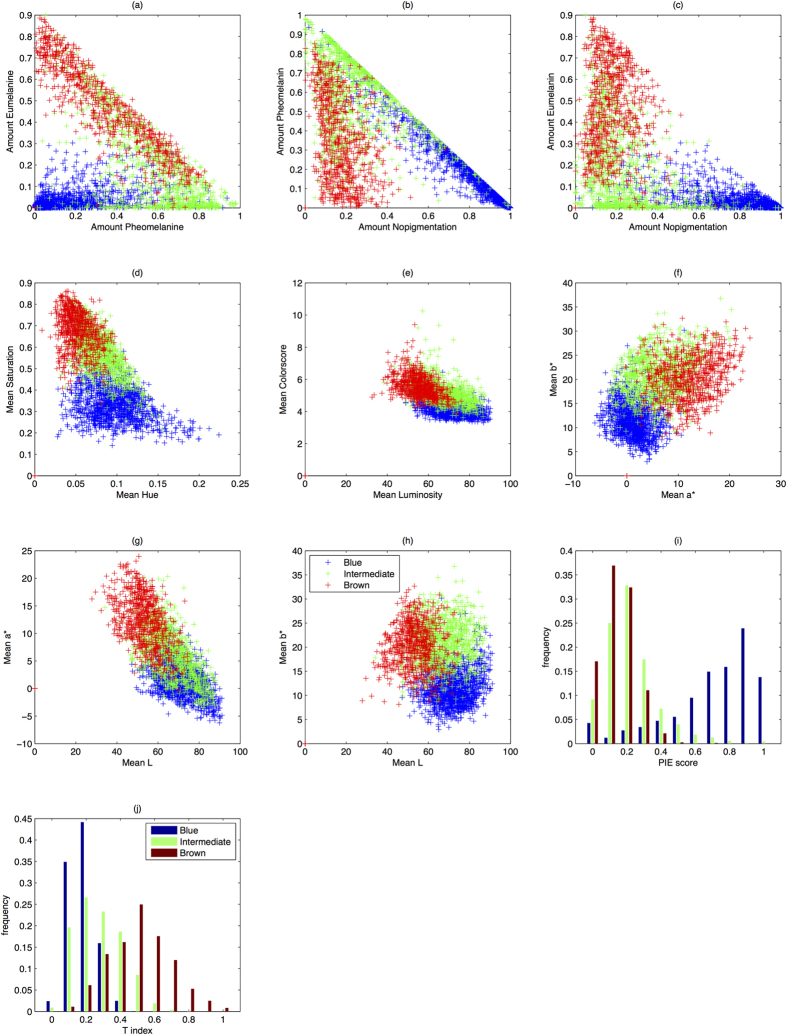
Manually categorized irises from the entire study population (N = 3087) into 3 eye colour categories blue (depicted in blue colour), intermediate (depicted in green colour), and brown (depicted in red colour) as arranged in the colour space of different quantification approaches for eye colour. Each data point depicts one individual iris categorized in one of the eye colour categories in the respective continuous colour space. Panels (a–h) depict the separation of the manually graded eye colours in two-dimensional continuous colour spaces, *e.g*. Pheomelanin vs. Eumelanin. Panels (i,j) depict the separation of manually graded eye colours on one-dimensional colour spaces, *i.e* PIE score and T-index respectively.

A good quantitative colour measure is expected to distinguish well among manually defined eye colour categories. To test for this, we plotted the manually derived eye colour categories (blue, intermediate, brown) in the different components of the described eye colour quantities (see Fig. 2). To demonstrate the quality in separating the distributions of two selected categories, we used the Hellinger distance (HD, Table 2). The HD describes the distance between two probability distributions, which is zero if both are perfectly overlapping and one in case they are entirely separated. Note that the PIE-score and T-index are only one-dimensional quantities. The manually graded eye colour categories blue and brown were best differentiated by the non-pigmentation/eumelanin-space generated by our new method (Fig. 2c, HD = 0.923), followed by the non-pigmentation/pheomelanin space from our method (Fig. 2b, HD = 0.911). The categories blue and brown can be nearly completely separated with our approach. The categories blue and intermediate were also best separated in the non-pigmentation/pheomelanin space from our method (Fig. 2b, HD = 0.743). However, brown and intermediate were best separated by the *L*-a* space (Fig. 2g, HD = 0.545), followed closely by the pheomelanin/eumelanin space from our approach (0.495). A large overlap of the intermediate category with both, blue and brown, was evident, likely caused by well-known difficulties in manual assignment of eye colours to the intermediate category.

Considering all pairs of categories, the colour score-luminance space performed least accurately in comparison to all other quantitative methods used. The summary of the a* and b* components most likely obscures important information about the different types of melanin. The one-dimensional PIE score and T-index measurements showed the weakest ability for the separation of intermediate eye colours from either brown (PIE score, HD = 0.228) or blue (T-index, HD = 0.323).

### Impact of improved eye colour phenotyping on strength of genetic eye colour association

Next, we investigated the impact of our eye colour phenotyping approach, as well as other previously used methods, on the strength of genetic eye colour association. For this, we used 12 SNPs from 11 genes highlighted in previous genetic eye colour studies mostly using categorical phenotypes^15^,^18^,^24^,^37^,^38^,^39^,^40^. We analysed these 12 SNPs in the same 3,087 individuals from whom we used the eye images for eye colour quantification. We employed partial correlation controlling for age, sex, and the sampling population (see Methods), and report the amount of genetically explained eye colour variance as measured by *R*^2^ (Table 3). Note that stronger association or higher effect size^41^ (as measured by *R*^2^ in our study) is beside the allele frequency - an important factor controlling for the power of discovering an unknown causal variant^42^. Due to the SNPs *HERC2* rs12913832 and *HERC2* rs1129038 being in very high linkage disequilibrium (LD) (*R*^2^ = 98%, *P* < 1e–300), we only describe the results for *HERC2* rs12913832 (for complete results, see Table 3). Among all quantitative measurements, the phenotypic eye colour variance explained by any of these 11 SNPs was highest for the amount of non-pigmentation as measured with our new approach (*HERC2* rs12913832 *R*^2^ = 49.7%, *P* < 1e–300) followed by *S (HERC2* rs12913832 *R*^2^ = 40.7%, *P* < e–300), and eumelanin measured with our approach (*HERC2* rs12913832 *R*^2^ = 32.1%, *P* = 1.3e–261), and H (*HERC2* rs12913832 *R*^2^ = 26.1%, *P* < 7e–205). The explained variance was considerably less of a* (HERC2 rs12913832 *R*^2^ = 15.4%, P = 1.e–300) and pheomelanin as measured with our approach (*HERC2* rs12913832 *R*^2^ = 14.6%, *P* = 1.8e–107). *HERC2* rs12913832 (and similarly the LD SNP *HERC2* rs1129038, Table 3) individually explained considerably more variation in continuous eye colour than any other SNP tested, which is in agreement with our previous study based on HS colour space^24^). This also is in line with previous categorical eye colour studies, where *HERC2* rs1129038 contributed most^12^,^15^,^38^.

In contrast to Liu *et al*.^22^, we could not replicate a statistically significant association of *NPLOC4* rs9894429, *DSCR9* rs2835630, and *LYST* rs3768056 (border-line significant association with *S, P* = 0.06) with any of the quantitative eye colour phenotypes tested including H and S used by Liu *et al*.^24^ (Table 3). The lack of association noted here could be explained by their small effect size together with the smaller sample size used here (N = 3,087) relative to the previous study (N = 5,951)^22^. Alternative factors could be image and individual age differences between EUREYE samples used here and the on average elder Rotterdam Study samples used previously (note that the effects are measured as single contributions to the phenotype, for combined analysis see Supplementary Table S2). Age has been previously shown to be a significant predictor for eye colour^24^.

### Epistatic effects detected with the detailed quantitative eye colour phenotypes

Next, we studied the impact of the detailed eye colour phenotyping as achieved with our new method, as well as previously used methods, on epistatic effects between pairs of SNPs that deviate from additivity while correcting for sex and age (see Methods and Table 4). For this, we ignored the interaction between *HERC2* rs1129038 and *HERC2* rs12913832 due to their strong LD (*R*^2^ = 0.98, *P* < 1e–300, Supplementary Table S3). We found two novel interactions between *SLC24A4* rs12896399 and *SLC45A2* rs16891982 strongest in pheomelanin (*P* = 8.7e–04) as well as *LYST* rs3768056 and *DSCR9* rs2835630 solely evident in eumelanin (*P* = 2.6e–2). Moreover, we confirmed several interaction pairs reported in previous studies using quantitative or categorical eye colour phenotypes, namely: *HERC2* rs12913832 and *SLC24A4* rs12896399 previously observed for S^24^ as well as blue vs. non-blue^43^, observed here in PIEscore (*P* = 2.3e–16), b* (*P* = 3.3e–4), S (*P* = 4.2e–3), non-pigmentation (*P* = 5.4e–13), and pheomelanin (*P* = 8e–9); *HERC2* rs12913832 and *SCL45A2* rs16891982 previously described in blue vs. non-blue^44^, observed here in *S (P* = 1.3e–12), pheomelanin (*P* = 1.2e–16), non-pigmentation (*P* = 1.8e–20), S (*P* = 1.3e–12), Colour score (*P* = 3.5e–7), PIEscore (*P* = 3.6e–21), and b* (*P* = 3.8e–18); *HERC2* rs12913832 and *TYPR1* rs1325127 previously described in hazel vs. non-hazel^43^ found only in pheomelanin (*P* = 7.8e–4); *HERC2* rs12913832 and *IRF4* rs12203592 previously observed in *H* and *S*^24^, found here in *H (P* = 1.9e–3), a* (*P* = 2.6e–4), and pheomelanin (*P* = 3.8e–3).

We exemplified the interaction between *HERC2* rs12913832 and *SLC45A2* rs16891982 in Supplementary Figure S2. From the marginal distribution of *HERC2* rs12913832 (SNP2 in Supplementary Figure S2) the dominant effect for allele T can be observed as the presence of a T allele strongly decreases the amount of non-pigmentation. The interaction partner *SLC45A2* rs16891982 shows a recessive behaviour for the G allele that increases the amount of non-pigmentation only for the homozygote genotype GG. From the combined distributions a masking effect of *HERC2* rs12913832 can be observed. The effect of the homozygote GG in *SLC45A2* rs16891982 becomes much more evident if *HERC2* rs12913832 is homozygote for CC (*R*^2^ = 0.037, *P* = 3.7e–12). The effect of *SLC45A2* rs16891982 on the amount of non-pigmentation is almost neutralized with *HERC2* rs12913832 heterozygote CT or homozygote TT (*R*^2^ = 0.003 *P* = 1.5e–02). Thus it appears that the allelic states of *SLC45A2* rs16891982 will lighten/darken only those irises that are already stated to be “blue” by *HERC2* rs12913832 = CC. A similar masking effect can be observed for the interaction pair *HERC2* rs12913832 and *SLC24A4* rs12896399 (Supplementary Figure S3). We similarly investigated the interaction between *HERC2* rs12913832 and *TYRP1* rs1325137 (Supplementary Figure S4) and observe a slight reinforcing effect of *TYRP1* rs1325137 on the amount of quantified pheomelanin. Having a C allele in *HERC2* rs12913832 decreases the amount of pheomelanin by the state of *TYRP1* rs1325137 (*R*^2^ = 0.06, *P* = 7.6e–3). In contrast, the state of *TYRP1* rs1325137 tends to increase the amount of pheomelanin if *HERC2* rs12913832 has at least one T allele (*R*^2^ = 0.02, *P* = 6.9e–2). Condensing all SNP-SNP interactions for certain quantitative eye colour estimates, the following SNP pairs were found to be most relevant: *IRF4* rs12203592 × *HERC2* rs12913832, *SLC24A4* rs12896399 × *SLC45A2* rs16891982, *HERC2* rs1291383) × *SLC24A4* rs12896399, *HERC2* rs12913832 × *TYRP1* rs1325127, *HERC2* rs12913832 × *SLC45A2* rs16891982. Consequently, we included them in the prediction analyses considered as a separate model.

### SNP-based prediction of quantitative eye colour phenotypes

We conducted a formal prediction analysis of continuous eye colour expressed by the various quantitative measures with (model 1, Supplementary Table S4) or without considering the SNP-SNP interaction terms previously described (model 2, Supplementary Table S5). For this, we divided our samples into a model building set (*N* = 2,087) and a model-validation set (*N* = 1,000). As prediction accuracy measure we used the percentage of phenotypic variance predicted by the respective model (mean coefficient of determination *R*^2^). Model 1 (without considering interactions) explained 52% of the phenotypic variance of non-pigmented area, 16% of pheomelanin, and 33% of eumelanin (Table 5), while *H* and *S* were predicted as 24% and 42%, respectively. PIE-score can be predicted as 45%, T-index as 31%, a* and b*, as 42% and 30% respectively. Model 2 (with considering interactions) provided increased prediction of 55% (*i.e*., by 3%) and 20% (*i.e*., by 4%) for non-pigmentation and pheomelanin, respectively, with eumelanin remaining at 33%, while for *S* the prediction increases to 44% (*i.e*., by 1%) and remained the same for *H* at 24%. Hence, by including the interaction terms in the prediction model, we observed an overall increase in the predictability of our newly derived quantitative eye colour measures. The strongest increase was seen for pheomelanin (*i.e*., by 4%) that notably is involved in intermediate eye colours. The following interactions were mostly relevant for the prediction of pheomelanin: *HERC2* rs12913832 and *SLC45A2* rs16891982 (beta = 0.73, *P* = 6.12e–14), *HERC2* rs12913832 and *SLC24A4* rs12896399 (beta = 0.32, *P* = 5.72e–9), as well as *IRF4* rs12203592 and *HERC2* rs12913832 (beta = 0.39, *P* = 9.56e–7), (see Supplementary Table S5).

Notably, when constraining the quantitative eye colour predictions on the 6 SNPs included in the IrisPlex system for categorical eye colour prediction^15^, we observed only a minor loss of information relative to the full 12-SNP model with interaction, and varying degrees of effect relative to the 12-SNP model without interaction (Supplementary Table S6). Based on the 6 IrisPlex SNPs with considering interaction (see Supplementary Table S7 for betas), we estimated 53% for the amount of non-pigmentation (2% less than the full 12-SNP model), 18% for the amount of pheomelanin (2% less), and 33% for the amount of eumelanin (same as the 12-SNP model). The observed slight increase of predictability of pheomelanin with the 12-SNPs relative to the 6 IrisPlex SNPs underlines the added value of the additional 6 SNPs in predicting non-blue and non-brown eye colours, which is notably lower than the gain of power by our novel quantification method.

**Figure 3:**
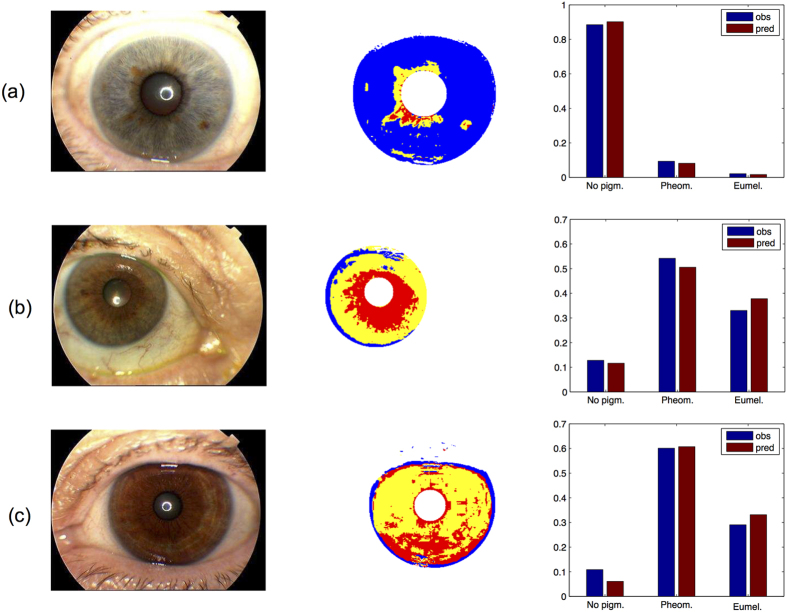
Example of three eyes that were manually categorized as blue (panel a), intermediate (panel b) or brown (panel c). The most left image panel represents the eye images taken with the Topcon camera system. The second from left image panel represents the segmented irises using our approach with the result of the supervised clustering into pheomelanin, eumelanin, and non-pigmented areas. The histograms on the most right show the observed (blue bars) and DNA-predicted (red bars) proportions of the respective types of eye pigment, i.e. eumelanin, pheomelanin and no pigmentation.

To demonstrate the potential of the 12 SNPs for predicting quantitative eye colour, in Fig. 3 we plotted the observed eye colour phenotypes as measured with our new approach, and their DNA-predictions from 12-SNP genotypes. We already find a very good prediction of the proportions of melanin-types, which is more informative with respect to iris colour than a plain category. Hence, intermediate iris colours can be visually predicted from DNA by the quantitative amounts of pheomelanin, and eumelanin, which would provide a better overall representation of eye colour rather than one single intermediate category. Knowledge about the distribution of the types of melanin on the iris will allow more realistic prediction of iris colour and structure, which can be hypothetically achieved by applying this approach to sub-areas of the iris as applied in Edwards *et al*.^27^.

## Conclusions

In summary, we have developed an automated computational approach that separates the iris from digital eye images and quantifies iris pigmentation by estimating digital equivalents of eumelanin, pheomelanin, and non-pigmentation. By applying this new approach to high-resolution eye imagery of thousands of Europeans, we demonstrated that it mostly outperformed previous eye colour quantification methods. When only low-resolution imagery is available that does not allow for a distinct clustering of pixels into melanin types, average *H* and *S* values are recommended for usage.

The power to detect a causal variant in a genome wide association study is known to depend mainly on its effect and frequency in the population^42^. By using these detailed quantitative eye colour phenotypes, we noted that SNPs previously involved in human eye colour variation showed stronger associations, revealed new and confirmed previously noted SNP-SNP interactions, and increased DNA-based prediction compared to quantitative eye colour phenotypes established by other methods. Overall, our findings imply that using our approach for detailed quantitative pigmentation phenotyping in future genome-wide association studies will likely deliver new pigmentation genes and new pigmentation predictive DNA variants, which is relevant for medical, evolutionary, and forensic genetics.

## Materials andMethods

### Ethics statement

The study was approved by national ethical committees and met the criteria of the Helsinki declaration. The ethics committees of each of the institutional review boards of the following collaborating eye study centres gave approval: Department of Epidemiology and Biostatistics, National Institute for Health Development, Tallinn, Estonia; Department of Ophthalmology, University of Bergen, School of Medicine, Bergen, Norway; Clinique Ophthalmologique, Universitaire De Creteil, Paris, France; Clinica Oculistica, Universita degli studi di Verona, Italy; Department of Ophthalmology, Aristotle University of Thessaloniki, School of Medicine, Thessaloniki, Greece; Dpto. Salud Publica Universidad Miguel Hernandez, Alicante, El Centro de Investigacion Biomedica en Red de Epidemiologıa y Salud Publica (CIBERESP), Elche, Spain; Faculty of Epidemiology & Population Health, London School of Hygiene & Tropical Medicine, London, United Kingdom. Study participants gave informed written consent. Participants were advised that all information would be kept confidential and no identifying information would be kept.

### Subjects, images, and genotyping

Eye images and DNA samples were collected as part of the EUREYE study. EUREYE is a population-based study of age related macular degeneration (AMD) in seven centers located across Europe. Participants were recruited from random sampling of the population aged over 65 years in Bergen (Norway), Tallinn (Estonia), Belfast (UK), Paris Creteil (France), Verona (Italy), Thessaloniki (Greece), and Alicante (Spain). Participants were interviewed by fieldworkers, underwent an eye examination including digital capture of the iris, and provided a blood sample for DNA analysis. A detailed description of the EUREYE study including image collection can be found elsewhere^38^,^45^,^46^. In brief, iris photography involved illumination of the anterior segment of each eye to show the colour of the iris with a flash intensity of 25–36 mW using a Topcon TRC 50 EX camera (http://www.topconmedical.com/categories/imaging-retinalcameras.htm) under normalized conditions. We manually excluded samples where the image quality was limited due to partially closed eyelids or largely dilated pupils that prevented a full view of the iris.

DNA samples were genotyped using the SNaPShot technology (Life Technologies) via a single multiplex assay targeting 12-eye colour SNPs. We used 6 SNPs that were previously identified to predict categorical eye colour in genome wide studies^12^,^24^,^37^) and which are included in the IrisPlex and HIrisPlex DNA test systems for categorical eye colour prediction^15^,^18^,^37^,^38^,^39^: *HERC2* rs12913832, *OCA2* rs1800407, *SLC24A4* rs12896399, *SLC45A2 (MATP*) rs16891982, *TYR* rs1393350, and *IRF4* rs12203592. The 6 additional SNPs used were identified to be eye colour predictive in previous studies^24^,^40^: rs2070959 (*UGT1A6*), rs9894429 (*NPLOC4*), rs1129038 (*HERC2*), rs3768056 (*LYST*), rs2835630 (*DSCR9*), and rs1325127 (*TYRP1*) (see Supplementary Table S1 for primer sequences). Our analyses in the present manuscript were based on 3087 samples with suitable iris pictures and complete genotype data available.

### Statistical analysis

Deviations from Hardy Weinberg equilibrium (HWE) were calculated using methods described elsewhere^47^. Among other causes, HWE can be violated because of genotyping error or population substructure. The latter is indicated by an increased informativeness of ancestry^48^. The statistical properties of the 12 SNPs from the 3087 samples are summarized in Supplementary Table S1. Three SNPs showed significant deviations from the Hardy-Weinberg equilibrium, namely *HERC2* rs1129038 (*P*_HWE_ = 8.25e–38), *HERC2* rs12913832 (*P*_HWE_ = 2.38e–37), and *SLC45A2* rs16891982 (*P*_HWE_ = 2e–16). In the absence of evidence for genotyping errors, we expect this being caused by the samples coming were from seven European populations as these three SNPs displayed elevated informativeness of ancestry (In) values (see Supplementary Table S1 for HWE and In values). Linkage disequilibrium (LD) between pairs of SNPs was estimated by means of *R*^2^. Regression analysis, Hellinger distance analysis, and interaction analysis were performed in Matlab (R2007a). For association and prediction analysis, linear models were fitted for both, individual SNPs and a combined model including all SNPs in Matlab (R2007a) while controlling for age, sex and population id. Presence of interactions between pairs of SNPs was tested by comparing nested linear models with and without the interaction term by means of an F-test. DNA-based eye colour prediction was achieved using general linear models *Y~b*_*0*_* *+* b*_*1*_*X*_*1*_* *+* b*_*2*_*X*_*2*_* *+* b*_*3*_*X*_*3*_* *+* b*_*4*_ *age *+* b*_*5*_ *sex *+* b*_*6*_ *pop*, where X_1_ and X_2_ denote the genotypes of SNP_1_ and SNP_2_ respectively and *X*_3_ denotes the interaction term on a multiplicative scale. Genotypes were coded additively as counts of minor alleles. P-value for the interaction term (*b*_3_) were used and corrected for multiple testing using Bonferroni correction for 12 interaction pairs (396 tests applied). Linear models were fitted to predict the quantitative amounts of melanin from genotypic variation. To study the importance of genetic interaction, we added the most important interaction identified to the predictor using only main effects of SNPs. Quality of prediction was assessed by means of adjusted coefficient of determination (*R*^2^) of the models. We used each colour quantification values separately as response variable. To account for over-fitting, we provide the mean *R*^2^ from cross validation as estimated from 100 randomized train/holdout experiments where 2/3 of the samples were used as a training set (*N* = 2087) and 1/3 as a validation set (*N* = 1000).

The computational method to perform statistical analyses and extract quantitative eye colour from digital images is available on request.

## Additional Information

**How to cite this article:** Wollstein, A. *et al*. Novel quantitative pigmentation phenotyping enhances genetic association, epistasis, and prediction of human eye colour. *Sci. Rep.* **7**, 43359; doi: 10.1038/srep43359 (2017).

**Publisher's note:** Springer Nature remains neutral with regard to jurisdictional claims in published maps and institutional affiliations.

## Supplementary Material

Supplementary Information

## Figures and Tables

**Table 1:** Pearson Correlation coefficients (*r*) between pairs of eye colour phenotype measures obtained with various methods in the European study population (N = 3,087), lower triangular matrix depicts corresponding P-values.

Eye colour measure	Nonpigmentation#	Pheomelanin#	Eumelanin#	Hue	Saturation	Colour score	L	a*	b*	PIE score	T-index
Nonpigmentation#	1	−0.64	−0.58	0.62	−0.74	0.57	−0.68	−0.66	0.93	−0.71	−0.61
Pheomelanin#	<1e–300	1	−0.06	−0.11	0.60	0.14	0.34	0.76	−0.68	0.29	0.13
Eumelanin#	1.12E–273	1.19E–03	1	−0.51	0.69	−0.52	0.71	0.38	−0.60	0.67	0.73
Hue	<1e–300	3.05E–09	1.10E–206	1	−0.37	0.73	−0.74	−0.07	0.51	−0.52	−0.86
Saturation	<1e–300	1.07E–299	<1e–300	2.57E–100	1	−0.17	0.81	0.89	−0.83	0.66	0.72
Colour score	1.12E–268	3.64E–14	1.43E–213	<1e–300	1.38E–21	1	−0.43	0.07	0.42	−0.47	0.71
L	<1e–300	1.38E–86	<1e–300	<1e–300	<1e–300	1.68E–139	1	0.53	−0.68	0.67	−0.76
a*	<1E–300	<1e–300	2.35E–104	3.65E–05	<1e–300	4.24E–05	8.11E–226	1	−0.74	0.50	0.97
b*	<1e–300	<1e–300	5.38E–303	4.20E–206	<1e–300	1.25E–130	<1e–300	<1e–300	1	−0.67	0.36
PIE score	<1e–300	6.12E–63	<1e–300	2.78E–217	<1e–300	1.16E–172	<1e–300	9.69E–197	<1e–300	1	−0.55
T-index	1.13E–300	1.18E–12	<1e–300	<1e–300	<1e–300	<1e–300	<1e–300	<1e–300	3.71E–92	5.99E–241	1

^#^Estimated with the new computational approach introduced here, for the methods used to measure the other eye colour scores, see text.

**Table 2:** Hellinger distances between pairs of colour categories from two dimensional colour subspaces (see Fig. 2).

	Interm.	Brown
**Amount pheomelanin vs**. **Amount Eumelanin (Fig. 2a)**		
Blue	0.720	0.851
Interm.	0	0.495
**Amount Nopigmentation vs**. **Amount Pheomelanin** (**Fig. 2b**)		
Blue	**0**.**743**	0.911
Interm.	0	0.491
**Amount Nopigmentation vs**. **Amount Eumelanin (Fig. 2c)**		
Blue	0.734	**0**.**923**
Interm.	0	0.480
**Mean Hue vs**. **Mean Saturation (Fig. 2d)**		
Blue	0.737	0.903
Interm.	0	0.470
**Mean Luminosity vs**. **Mean Colourscore (Fig. 2e)**		
Blue	0.639	0.814
Interm.	0	0.473
Mean a* vs mean b* (Fig. 2f)		
Blue	0.726	0.888
Interm.	0	0.495
**Mean L vs**. Mean a* (**Fig. 2g**)		
Blue	0.461	0.838
Interm.	0	**0**.**545**
**Mean L vs**. Mean b* (**Fig. 2h**)		
Blue	0.697	0.809
Interm.	0	0.491
**PIE score (Fig. 2i)**		
Blue	0.668	0.794
Interm.	0	0.228
**T-index (Fig. 2j)**		
Blue	0.323	0.733
Interm.	0	0.491

The higher the value close to one, the better is separability of the respective clusters in the quantitative colour space. Bold values denote the components that separate pairs of categories best.

**Table 3:** Single associations of 12 SNPs previously involved in human eye colour variation with eye colour phenotype measures in the European study population (N = 3,087) controlled for age and sex in *R* ^2^ (P-values).

SNP	Gene	Nopigm.#	Pheomelanin#	Eumelanin^#^	Saturation	Hue	Colour score	L	a*	b*	PIE score	T-index
rs1800407	*OCA2*	**1**.**0** (**3**.**7e**–**08**)	1.0 (3.8e–08)	0.1 (7.2e–02)	0.5 (1.5e–04)	0.1 (1.7e–01)	0.0 (3.1e–03)	0.0 (6.2e–01)	0.0 (1.2e–01)	0.0 (5.3e–07)	0.0 (1.8e–07)	0.0 (7.3e–01)
rs2070959	*UGT1A6*	0.2 (1.4e–02)	0.0 (2.7e–01)	0.3 (1.7e–03)	**0**.**4** (**9**.**0e**–**04**)	0.3 (3.1e–03)	0.0 (1.1e–03)	0.0 (8.9e–02)	0.0 (1.6e–04)	0.0 (7.5e–03)	0.0 (2.1e–03)	0.0 (3.2e–04)
rs9894429	*NPLOC4*	0.0 (4.0e–01)	0.0 (5.0e–01)	0.0 (3.0e–01)	0.1 (1.6e–01)	0.0 (9.3e–01)	0.0 (1.9e–01)	0.0 (9.7e–01)	0.0 (5.8e–01)	0.0 (1.8e–01)	0.0 (5.0e–01)	0.0 (7.0e–01)
rs1129038^$^	*HERC2*	**49**.**2** (<**1e–300**)	14.4 (7.4e–106)	32.3 (2.4e–263)	40.7 (<1e–300)	25.7 (2.6e–201)	4.4 (1.2e–160)	2.3 (3.5e–117)	15.3 (<1e–300)	7.9 (4.2e–224)	18.1 (0.0e + 00)	11.3 (4.7e–269)
rs12203592	*IRF4*	1.2 (5.2e–10)	0.0 (6.0e–01)	**2**.**9** (**3**.**3e**–**21**)	1.9 (7.2e–15)	1.2 (5.4e–10)	0.0 (2.2e–10)	0.0 (1.2e–11)	0.0 (5.9e–12)	0.0 (3.0e–10)	0.0 (2.7e–14)	0.0 (9.3e–12)
rs1393350	*TYR*	0.6 (1.4e–05)	0.4 (2.6e–04)	0.3 (1.5e–03)	**0**.**8** (**8**.**9e**–**07**)	0.2 (1.2e–02)	0.0 (3.7e–05)	0.0 (1.8e–01)	0.0 (2.5e–04)	0.0 (1.3e–05)	0.0 (3.5e–07)	0.0 (3.7e–03)
rs12913832^$^	*HERC2*	**49**.**7** (**<1e–300**)	14.6 (1.8e–107)	32.1 (1.3e–261)	40.7 (0.0e + 00)	26.1 (7.1e–205)	4.3 (9.1e–159)	2.4 (1.2e–119)	15.4 (<1e–300)	8.0 (7.9e–225)	18.3 (0.0e + 00)	11.4 (1.2e–270)
rs12896399	*SLC24A4*	**3**.**9** (**9**.**2e**–**29**)	1.8 (1.1e–13)	1.3 (1.8e–10)	2.4 (6.1e–18)	1.9 (1.2e–14)	0.0 (3.1e–09)	0.0 (2.6e–09)	0.0 (3.9e–15)	0.0 (1.6e–15)	0.1 (3.2e–28)	0.0 (7.2e–12)
rs3768056	*LYST*	0.1 (2.1e–01)	0.0 (3.5e–01)	0.0 (2.8e–01)	0.1 (6.2e–02)	0.0 (3.8e–01)	0.0 (1.2e–01)	0.0 (7.2e–01)	0.0 (1.4e–01)	0.0 (8.7e–02)	0.0 (1.3e–01)	0.0 (3.1e–01)
rs2835630	*DSCR9*	0.0 (7.6e–01)	0.0 (9.3e–01)	0.0 (2.4e–01)	0.0 (2.4e–01)	0.0 (2.5e–01)	0.0 (1.7e–01)	0.0 (6.7e–01)	0.0 (1.0e–01)	0.0 (5.2e–01)	0.0 (5.7e–01)	0.0 (1.0e–01)
rs16891982	*SLC45A2*	**4**.**1** (**5**.**2e**–**30**)	0.7 (2.4e–06)	2.7 (2.9e–20)	2.7 (2.9e–20)	3.5 (2.0e–25)	0.0 (7.0e–10)	0.0 (8.3e–16)	0.2 (1.4e–28)	0.0 (7.0e–13)	0.1 (1.1e–26)	0.2 (2.2e–28)
rs1325127	*TYRP1*	0.9 (7.5e–08)	0.1 (4.2e–02)	**1**.**0** (**2**.**7e**–**08**)	1.0 (3.2e–08)	0.9 (1.2e–07)	0.0 (1.4e–04)	0.0 (5.7e–05)	0.0 (5.9e–10)	0.0 (1.1e–04)	0.0 (3.4e–07)	0.0 (1.0e–01)

*R*^2^ values are provided in percentages, ^#^estimated with the new computational approach introduced here, Bold values emphasize the strongest association within the quantitative phenotypes per SNP.^$^ The SNPs rs1129038 and rs12913832 are in strong LD (*R*^2^ = 0.99) and have no independent effects on the phenotype. Please refer to Supplementary Table S2 for betas.

**Table 4:** Statistically significant interaction between pairs of SNPs from different pigmentation genes in the European study population (N = 3,087).

Interacting genes	Eye colour measure	SNP1 Chr	SNP2 Chr	P-value*
*HERC2 x SLC24A4*	PIEscore	rs12913832 15	rs12896399 14	2.37e–16
*HERC2 x SLC24A4*	b*	rs12913832 15	rs12896399 14	3.34e–04
*HERC2 x SLC24A4*	Saturation	rs12913832 15	rs12896399 14	4.20e–03
*HERC2 x SLC24A4*	Non-pigm.	rs12913832 15	rs12896399 14	5.49e–13
*HERC2 x SLC24A4*	Pheomel.	rs12913832 15	rs12896399 14	8.37e–09
*HERC2 x SLC45A2*	Pheomel.	rs12913832 15	rs16891982 5	1.26e–16
*HERC2 x SLC45A2*	Saturation	rs12913832 15	rs16891982 5	1.30e–12
*HERC2 x SLC45A2*	Non-pigm.	rs12913832 15	rs16891982 5	1.48e–20
*HERC2 x SLC45A2*	Colour score	rs12913832 15	rs16891982 5	3.52e–07
*HERC2 x SLC45A2*	PIEscore	rs12913832 15	rs16891982 5	3.65e–21
*HERC2 x SLC45A2*	b*	rs12913832 15	rs16891982 5	3.83e–18
*HERC2 x TYRP1*	Pheomel.	rs12913832 15	rs1325127 9	7.84e–04
*IRF4 x HERC2*	Hue	rs12203592 6	rs12913832 15	1.95e–03
*IRF4 x HERC2*	a*	rs12203592 6	rs12913832 15	2.67e–04
*IRF4 x HERC2*	Pheomel.	rs12203592 6	rs12913832 15	3.88e–03
*LYST x DSCR9*	Eumel.	rs3768056 1	rs2835630 21	2.77e–02
*SLC24A4 x SLC45A2*	b*	rs12896399 14	rs16891982 5	1.80e–02
*SLC24A4 x SLC45A2*	Saturation	rs12896399 14	rs16891982 5	2.48e–02
*SLC24A4 x SLC45A2*	PIEscore	rs12896399 14	rs16891982 5	3.22e–02
*SLC24A4 x SLC45A2*	Colour score	rs12896399 14	rs16891982 5	4.50e–03
*SLC24A4 x SLC45A2*	Pheomel.	rs12896399 14	rs16891982 5	8.72e–04

*HERC2* rs1129038 was excluded due to strong LD with *HERC2* rs12913832, *Significance threshold according to Bonferroni correction: <2.5e–5, ^#^estimated with the new computational approach introduced here.

**Table 5:** Mean coefficient of determination (*R* ^2^ in %) of different quantitative eye colour measures using 12 SNPs* in the European study population (N = 3,087).

Eye colour measure	R^2^ from Prediction Model 1 Without SNP-SNP interaction**	R^2^ from Prediction Model 2 With SNP-SNP interaction**
Non-pigm.	51.99 (48.68, 54.86)	54.79 (51.54, 57.83)
Pheomel.	15.56 (12.55, 19.23)	19.63 (16.54, 22.45)
Eumel.	32.85 (29.07, 36.25)	32.52 (28.89, 36.71)
Hue	23.55 (20.33, 26.25)	24.15 (21.02, 27.83)
Saturation	43.38 (38.76, 47.41)	44.25 (40.09, 48.77)
Colour score	22.46 (17.84, 26.86)	22.79 (18.74, 27.50)
L	16.56 (13.53, 19.98)	16.88 (13.18, 20.03)
a*	41.77 (38.62, 45.18)	41.99 (38.30, 46.34)
b*	29.64 (25.34, 33.39)	32.03 (28.51, 35.76)
PIE score	45.17 (41.98, 48.79)	48.60 (45.81, 51.47)
T-index	31.40 (27.74, 34.84)	32.55 (28.92, 35.93)

*See Table 1 for the SNPs. **Values in brackets denote the 5% and 95% quantile from crossvalidation respectively. ^#^Estimated with the new computational approach introduced here.
