# Supplementary material for: Novel quantitative pigmentation phenotyping enhances genetic association, epistasis, and prediction of human eye colour
Source: Sci Rep. 2017 Feb 27;7:43359. doi: 10.1038/srep43359 (PMC5327401; doi:10.1038/srep43359)
Supplement: Supplementary Information [file srep43359-s1.doc]

**Novel quantitative pigmentation phenotyping enhances genetic association, epistasis, and prediction of human eye colour**

Andreas Wollstein^1,2,3^,*, Susan Walsh^1,4^, Fan Liu^1,5^, Usha Chakravarthy^6^, Mati Rahu^7^, Johan H. Seland^8^, Gisele Soubrane^9^, Laura Tomazzoli^10^, Fotis Topouzis^11^, Johannes R. Vingerling^12^, Jesus Vioque^13^, Stefan Böhringer^2^, Astrid E. Fletcher^14^, and Manfred Kayser^1^*

**Supplementary information**


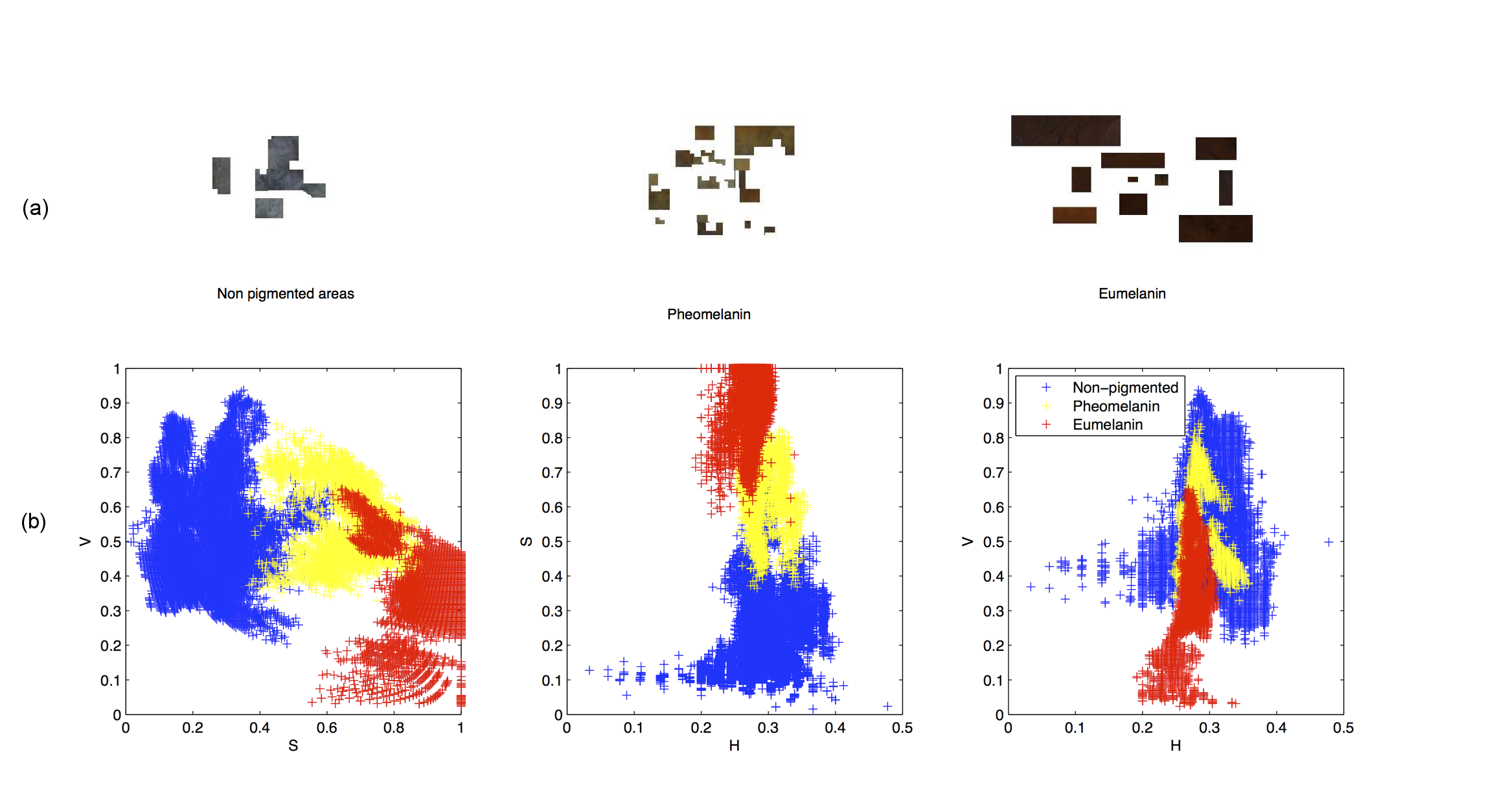


Figure S1: Example images of manually selected pigmentation types and their respective distribution in the in HSV space. Panel (a) depicts manually selected samples from ten random irises images used for training that represent pheomelanin, eumelanin, or absence of pigmentation. Panel (b) depicts the distribution of color distribution of the latter described areas as projections from HSV color space. Different colors depict the different types of pigmentation with blue indicating non-pigmentation red indicating eumelanin, and yellow indicating pheomelanin; each dot represents the pixel of a pigmentation class in the respective color space.

Figure S2. Depicted are the distribution of non-pigmentation represented as box plots conditioned on the combined genotypes for the interacting SNPs rs16891982 (*SLC45A2*) and rs12913832 (*HERC2*). The fourth row and column depicts the marginal distributions conditioned on the genotypes of the respective (single) SNPs. The dotted red line denotes the median effect from the respective genotypes of the two SNPs as expected from additivity.

Figure S3. Depicted are the non-pigmentation distribution represented as box plots conditioned on the combined genotypes for the interaction pair rs12896399 (*SLC24A4*) and rs12913832 (*HERC2*). See Supplementary Figure S2 for further detail.

Figure S4. Depicted are the pheomelanin distribution represented as box plots conditioned on the combined genotypes for the interaction pair rs12913832 (*HERC2*) and rs1325137 (*TYRP1*). See Supplementary Figure S2 for further detail.

Table S1: Genotyped SNPs with single site statistics in the study population

| **No.** | **SNP** | **Gene** | **Ch/s** | **pos_hg18** | **P-Hwe** | **He_obs** | **MAF** | **In** | **PCR primers** | |  |  | **SBE primers** | **Sequence** |
| --- | --- | --- | --- | --- | --- | --- | --- | --- | --- | --- | --- | --- | --- | --- |
| 1 | rs1800407 | *OCA2* | 15 | 25903913 | 5.31E-01 | 0.128 | 0.431 | 0.009 | F | TGAAAGGCTGCCTCTGTTCT | R | CGATGAGACAGAGCATGATGA | **SBE-F** | TTTTGCATACCGGCTCTCCC |
| 2 | rs2070959 | *UGT1A6* | 2 | 226402180 | 5.36E-01 | 0.449 | 0.168 | 0.004 | F | ATTTGGGCCTACCATCTGTG | R | TTGTGTAGCACCTGGGAATG | **SBE-F** | TTTTTTTTCGTGTTCCCTGGAGCAT |
| 3 | rs9894429 | *NPLOC4* | 17 | 75043296 | 6.02E-01 | 0.499 | 0.051 | 0.001 | F | TGTTGCTGTGATCCGCTTC | R | AGGACCTCACTAGGCTGTGC | **SBE-F** | TTTTTTTTTTTTGATCCGCTTCACTCCATC |
| 4 | rs1129038 | *HERC2* | 15 | 26030454 | 8.25E-38 | 0.374 | 0.118 | 0.098 | F | TCCTTTGCTTCGGACTCTACA | R | ACACCAGGCAGCCTACAGTC | **SBE-R** | TTTTTTTTTTTTTTTTTTCAGTCTACACAGCAGCGAG |
| 5 | rs12203592 | *IRF4* | 6 | 341321 | 2.57E-03 | 0.214 | 0.371 | 0.046 | F | ACAGGGCAGCTGATCTCTTC | R | GCTAAACCTGGCACCAAAAG | **SBE-F** | TTTTTTTTTTTTTTTTTTTTTTTTTATTTGGTGGGTAAAAGAAGG |
| 6 | rs1393350 | *TYR* | 11 | 88650694 | 6.95E-01 | 0.368 | 0.254 | 0.002 | F | GCGTGCATATCCACCAACT | R | TGTTTGTATCTGGGAAGGTGAA | **SBE-R** | TTTTTTTTTTTTTTTTTTTTTTTTTTATTTGTAAAAGACCACACAGATTT |
| 7 | rs12913832 | *HERC2* | 15 | 26039213 | 2.38E-37 | 0.374 | 0.12 | 0.072 | F | GAATTTGTTCTTCATGGCTCTCT | R | GGCCCCTGATGATGATAGC | **SBE-R** | TTTTTTTTTTTTTTTTTTTTTTTTTTTTTTTTTTTTTTGCGTGCAGAACTTGACA |
| 8 | rs12896399 | *SLC24A4* | 14 | 91843416 | 5.70E-02 | 0.46 | 0.112 | 0.006 | F | CTGGCGATCCAATTCTTTGT | R | CTTAGCCCTGGGTCTTGATG | **SBE-F** | TTTTTTTTTTTTTTTTTTTTTTTTTTTTTTTTTTTTATCTTTAGGTCAGTATATTTTGGG |
| 9 | rs3768056 | *LYST* | 1 | 233974448 | 2.18E-01 | 0.374 | 0.258 | 0.001 | F | GGATCTACAGAGCTGTTTCTCTGC | R | TGTGCAACAGACTCCCAGAC | **SBE-R** | TTTTTTTTTTTTTTTTTTTTTTTTTTTTTTTTTTTTTTTTTACCATGATTATCACATATACAGCA |
| 10 | rs2835630 | *DSCR9* | 21 | 37443712 | 1.65E-02 | 0.479 | 0.032 | 0.002 | F | CCCTCTTTTAGTGGTCTTAACTATTCC | R | TGCCACAAGATATTTGGGTTG | **SBE-R** | TTTTTTTTTTTTTTTTTTTTTTTTTTTTTTTTTTTTTTTTTTTTTTTGGAAAGCTGACTAATTTACAGAG |
| 11 | rs16891982 | *SLC45A2* | 5 | 33987450 | 2.03E-16 | 0.127 | 0.418 | 0.077 | F | TCCAAGTTGTGCTAGACCAGA | R | CGAAAGAGGAGTCGAGGTTG | **SBE-R** | TTTTTTTTTTTTTTTTTTTTTTTTTTTTTTTTTTTTTTTTTTTTTTTTTTTTTTTTTTTTTGGATGTTGGGGCTT |
| 12 | rs1325127 | *TYRP1* | 9 | 12658328 | 2.07E-01 | 0.451 | 0.141 | 0.003 | F | TCTGTTGTTAGCCTACCTAGATGTTT | R | AAACATAAAAACATGATGGAACACA | **SBE-F** | TTTTTTTTTTTTTTTTTTTTTTTTTTTTTTTTTTTTTTTTTTTTTTTTTTTTTTATGTTGTTAGCCTACCTAGATGTTTA |

Abbrevations used: pos_hg18: position on reference genome hg18, P-Hwe: P-value for being out of Hardy Weinberg equilibrium, He_obs: observed heterozygosity, MAF: minor allele frequency, In: informativeness of ancestry (Rosenberg et al. 2003).

Table S2. Betas and standard errors in brackets from multivariate analysis including the most relevant interactions, as well as age and sex.

| **Beta (Pval)** | **Nonpigm.** | **Pheomel.** | **Eumel.** | **Hue** | **Saturation** | **Colorsc.** | **L** | **a*** | **b*** | **PIEscore** | **T-index** |
| --- | --- | --- | --- | --- | --- | --- | --- | --- | --- | --- | --- |
| mean | -7.45 (2.71e-07) | 4.50 (3.07e-03) | 3.96 (6.08e-02) | -0.73 (3.66e-01) | 2.66 (2.07e-03) | 0.77 (2.82e-01) | 0.25 (7.71e-01) | -0.21 (8.15e-01) | 2.57 (1.57e-03) | -7.30 (9.63e-07) | -1.04 (2.74e-01) |
| rs1800407 | -0.21 (5.88e-03) | 0.04 (5.92e-01) | 0.45 (5.13e-05) | -0.09 (3.13e-02) | 0.18 (7.74e-05) | 0.06 (9.57e-02) | -0.17 (1.86e-04) | 0.18 (6.32e-05) | -0.04 (3.49e-01) | -0.15 (5.98e-02) | 0.14 (4.30e-03) |
| rs2070959 | -0.06 (1.41e-01) | 0.01 (8.27e-01) | 0.07 (2.32e-01) | -0.00 (9.25e-01) | 0.03 (1.79e-01) | -0.01 (6.79e-01) | 0.00 (9.56e-01) | 0.04 (1.03e-01) | -0.01 (7.49e-01) | -0.05 (1.99e-01) | 0.01 (7.51e-01) |
| rs9894429 | 0.05 (1.51e-01) | -0.00 (9.19e-01) | -0.02 (7.55e-01) | 0.01 (6.08e-01) | -0.05 (2.44e-02) | -0.03 (1.13e-01) | 0.02 (5.05e-01) | 0.01 (7.93e-01) | -0.04 (5.94e-02) | 0.04 (3.54e-01) | -0.04 (9.23e-02) |
| rs1129038 | 0.49 (8.60e-02) | 0.14 (6.32e-01) | -1.19 (4.18e-03) | 0.12 (4.52e-01) | -0.27 (1.12e-01) | -0.08 (5.62e-01) | 0.27 (1.18e-01) | -0.30 (8.27e-02) | -0.05 (7.35e-01) | 0.44 (1.27e-01) | -0.29 (1.16e-01) |
| rs12203592 | 0.68 (5.21e-07) | -0.73 (3.03e-07) | -0.19 (3.33e-01) | -0.12 (1.09e-01) | -0.07 (3.97e-01) | -0.05 (4.99e-01) | 0.13 (1.25e-01) | 0.20 (1.42e-02) | -0.24 (1.92e-03) | 0.79 (2.68e-08) | 0.36 (5.19e-05) |
| rs1393350 | 0.23 (1.07e-07) | -0.18 (6.61e-05) | -0.14 (2.69e-02) | 0.00 (9.29e-01) | -0.04 (1.30e-01) | -0.00 (8.61e-01) | 0.03 (1.82e-01) | -0.05 (5.94e-02) | -0.06 (1.59e-02) | 0.19 (1.50e-05) | -0.04 (1.93e-01) |
| rs12913832 | 2.26 (3.87e-08) | -2.57 (2.91e-09) | -0.83 (1.64e-01) | -0.18 (4.22e-01) | -0.31 (2.01e-01) | -0.20 (3.27e-01) | -0.19 (4.48e-01) | 0.21 (4.00e-01) | -0.81 (4.50e-04) | 2.36 (2.49e-08) | 0.70 (1.01e-02) |
| rs12896399 | 0.45 (1.54e-01) | -0.26 (4.36e-01) | -0.75 (1.05e-01) | 0.02 (9.27e-01) | -0.23 (2.18e-01) | -0.06 (6.97e-01) | 0.15 (4.39e-01) | 0.38 (5.23e-02) | 0.15 (3.98e-01) | 0.40 (2.22e-01) | 0.23 (2.69e-01) |
| rs3768056 | 0.05 (2.41e-01) | -0.03 (5.21e-01) | -0.10 (1.18e-01) | -0.00 (9.12e-01) | -0.06 (3.42e-02) | 0.00 (9.22e-01) | -0.00 (9.90e-01) | -0.03 (2.11e-01) | -0.04 (7.74e-02) | 0.02 (6.17e-01) | -0.03 (3.31e-01) |
| rs2835630 | 0.06 (1.16e-01) | -0.03 (4.43e-01) | -0.08 (1.34e-01) | 0.04 (6.31e-02) | -0.03 (1.95e-01) | -0.01 (5.87e-01) | 0.04 (6.76e-02) | -0.01 (6.91e-01) | -0.01 (7.20e-01) | 0.07 (5.89e-02) | -0.03 (1.85e-01) |
| rs16891982 | 1.63 (2.00e-09) | -1.32 (3.91e-06) | -0.80 (4.20e-02) | 0.08 (6.12e-01) | -0.70 (1.64e-05) | -0.24 (7.68e-02) | 0.14 (4.07e-01) | -0.18 (2.77e-01) | -0.64 (2.62e-05) | 1.39 (7.58e-07) | -0.08 (6.65e-01) |
| rs1325127 | 0.43 (1.66e-05) | -0.40 (1.61e-04) | -0.26 (7.52e-02) | 0.07 (1.93e-01) | -0.03 (6.62e-01) | -0.05 (2.95e-01) | -0.02 (7.47e-01) | -0.03 (5.76e-01) | -0.13 (2.07e-02) | 0.39 (1.50e-04) | -0.04 (5.24e-01) |
| rs12203592 x rs12913832 | -0.26 (6.59e-04) | 0.39 (1.06e-06) | -0.07 (5.11e-01) | 0.10 (2.33e-02) | -0.03 (4.97e-01) | -0.00 (9.73e-01) | 0.00 (9.49e-01) | -0.16 (4.65e-04) | 0.10 (2.10e-02) | -0.30 (1.05e-04) | -0.26 (2.89e-07) |
| rs12896399 x rs16891982 | 0.14 (1.60e-01) | -0.21 (4.52e-02) | 0.02 (9.03e-01) | 0.04 (4.53e-01) | -0.00 (9.56e-01) | -0.01 (9.08e-01) | -0.05 (4.38e-01) | -0.14 (1.81e-02) | -0.12 (2.64e-02) | 0.16 (1.31e-01) | -0.06 (3.86e-01) |
| rs12913832 x rs12896399 | -0.31 (1.49e-09) | 0.32 (4.85e-09) | 0.26 (6.34e-04) | -0.04 (1.61e-01) | 0.08 (7.57e-03) | 0.03 (3.08e-01) | 0.00 (9.49e-01) | -0.01 (6.95e-01) | 0.07 (1.20e-02) | -0.32 (2.72e-09) | -0.08 (2.68e-02) |
| rs12913832 x rs1325127 | -0.18 (3.98e-04) | 0.20 (1.28e-04) | 0.07 (3.81e-01) | -0.01 (6.98e-01) | -0.03 (3.17e-01) | 0.02 (4.94e-01) | 0.02 (4.17e-01) | 0.00 (9.66e-01) | 0.04 (1.35e-01) | -0.17 (1.47e-03) | -0.00 (9.83e-01) |
| rs12913832 x rs16891982 | -0.72 (6.68e-15) | 0.72 (8.00e-14) | 0.24 (7.24e-02) | -0.02 (6.59e-01) | 0.27 (9.71e-07) | 0.10 (3.24e-02) | -0.00 (9.80e-01) | 0.11 (5.43e-02) | 0.30 (8.73e-09) | -0.64 (2.11e-11) | 0.02 (7.61e-01) |
| age | 0.00 (1.33e-01) | -0.00 (6.06e-01) | -0.01 (2.74e-02) | 0.00 (6.74e-01) | -0.00 (4.06e-02) | -0.00 (3.95e-01) | 0.00 (4.84e-01) | -0.00 (3.10e-01) | -0.00 (2.76e-01) | 0.00 (3.76e-01) | -0.00 (5.55e-01) |
| sex | -0.14 (9.51e-03) | 0.13 (1.55e-02) | 0.08 (2.89e-01) | 0.05 (6.56e-02) | 0.05 (1.44e-01) | 0.03 (2.76e-01) | -0.03 (2.94e-01) | -0.00 (9.18e-01) | 0.09 (1.86e-03) | -0.07 (2.13e-01) | -0.01 (7.83e-01) |
| Total R^2* [%] | 54.55 (51.67,57.26) | 19.44 (16.52,21.88) | 32.89 (29.13,36.27) | 24.02 (20.53,27.35) | 44.67 (40.34,48.95) | 23.34 (18.77,27.44) | 16.90 (13.56,20.81) | 41.88 (38.41,45.32) | 31.98 (27.74,35.82) | 48.49 (45.64,50.93) | 32.00 (28.10,35.78) |

*Total R^2 was estimated from cross validation. Numbers in brackets denote 5% and 95% quantile respectively.

Table S3: Measure of linkage disequilibrium between paris of SNPs. The upper and lower triangular matrices maintain the R^2 value and p values, respectively.

| **R2 / Pval** | **rs1800407** | **rs2070959** | **rs9894429** | **rs1129038** | **rs12203592** | **rs1393350** | **rs12913832** | **rs12896399** | **rs3768056** | **rs2835630** | **rs16891982** | **rs1325127** |
| --- | --- | --- | --- | --- | --- | --- | --- | --- | --- | --- | --- | --- |
| **rs1800407** | 1 | 7.12E-04 | 3.29E-04 | 3.86E-02 | 6.61E-04 | 3.41E-04 | 3.72E-02 | 1.45E-04 | 6.56E-04 | 1.31E-04 | 5.59E-05 | 3.09E-06 |
| **rs2070959** | 1.38E-01 | 1 | 1.13E-04 | 1.94E-03 | 9.39E-04 | 3.73E-04 | 1.83E-03 | 1.29E-04 | 1.41E-03 | 4.30E-04 | 3.44E-06 | 1.69E-03 |
| **rs9894429** | 3.14E-01 | 5.55E-01 | 1 | 1.49E-04 | 1.75E-05 | 2.43E-05 | 1.26E-04 | 8.06E-06 | 1.13E-03 | 9.78E-04 | 1.33E-04 | 1.73E-05 |
| **rs1129038** | 3.13E-28 | 1.44E-02 | 4.98E-01 | 1 | 1.07E-02 | 1.79E-03 | 9.84E-01 | 1.34E-02 | 1.27E-05 | 3.28E-05 | 3.06E-02 | 9.13E-03 |
| **rs12203592** | 1.53E-01 | 8.87E-02 | 8.16E-01 | 7.77E-09 | 1 | 1.19E-03 | 1.10E-02 | 1.38E-03 | 1.48E-04 | 1.42E-04 | 5.77E-03 | 1.76E-04 |
| **rs1393350** | 3.05E-01 | 2.83E-01 | 7.84E-01 | 1.88E-02 | 5.51E-02 | 1 | 2.10E-03 | 2.41E-04 | 1.04E-04 | 1.14E-05 | 1.80E-03 | 2.55E-04 |
| **rs12913832** | 2.90E-27 | 1.74E-02 | 5.33E-01 | 0.00E+00 | 5.03E-09 | 1.09E-02 | 1 | 1.38E-02 | 8.50E-05 | 1.58E-06 | 3.17E-02 | 8.72E-03 |
| **rs12896399** | 5.03E-01 | 5.28E-01 | 8.75E-01 | 1.12E-10 | 3.89E-02 | 3.89E-01 | 6.10E-11 | 1 | 1.12E-07 | 7.40E-05 | 2.92E-03 | 2.23E-04 |
| **rs3768056** | 1.55E-01 | 3.66E-02 | 6.15E-02 | 8.43E-01 | 4.99E-01 | 5.72E-01 | 6.09E-01 | 9.85E-01 | 1 | 1.73E-05 | 9.73E-05 | 3.49E-06 |
| **rs2835630** | 5.24E-01 | 2.49E-01 | 8.24E-02 | 7.50E-01 | 5.09E-01 | 8.51E-01 | 9.44E-01 | 6.33E-01 | 8.17E-01 | 1 | 9.51E-06 | 2.12E-05 |
| **rs16891982** | 6.78E-01 | 9.18E-01 | 5.22E-01 | 1.30E-22 | 2.40E-05 | 1.83E-02 | 1.96E-23 | 2.65E-03 | 5.84E-01 | 8.64E-01 | 1 | 1.07E-03 |
| **rs1325127** | 9.22E-01 | 2.22E-02 | 8.17E-01 | 1.04E-07 | 4.61E-01 | 3.75E-01 | 2.03E-07 | 4.07E-01 | 9.17E-01 | 7.98E-01 | 6.93E-02 | 1 |

Table S4. Betas and P-values from prediction model 1. Significant contributions (P<0.01) have been marked in bold.

| **Beta (Pval)** | **Nonpigm** | **Pheomel.** | **Eumel.** |
| --- | --- | --- | --- |
| mean | -1.59 (1.93e-01) | -1.23 (3.41e-01) | 0.98 (5.73e-01) |
| rs1800407 | -0.15 (4.65e-02) | -0.02 (8.35e-01) | **0.42 (1.23e-04)** |
| rs2070959 | -0.06 (1.11e-01) | 0.01 (7.43e-01) | 0.08 (1.95e-01) |
| rs9894429 | 0.07 (8.81e-02) | -0.02 (6.51e-01) | -0.02 (7.68e-01) |
| rs1129038 | 0.52 (7.27e-02) | 0.10 (7.42e-01) | **-1.18 (4.51e-03)** |
| rs12203592 | **0.27 (1.09e-06)** | -0.10 (8.25e-02) | **-0.31 (5.66e-05)** |
| rs1393350 | **0.23 (1.82e-07)** | **-0.18 (1.01e-04)** | -0.15 (1.95e-02) |
| rs12913832 | **-1.01 (5.17e-04)** | **0.94 (2.19e-03)** | 0.36 (3.86e-01) |
| rs12896399 | **0.31 (1.51e-15)** | **-0.31 (5.89e-14)** | **-0.25 (4.70e-06)** |
| rs3768056 | 0.05 (2.46e-01) | -0.03 (5.51e-01) | -0.10 (1.13e-01) |
| rs2835630 | 0.05 (1.64e-01) | -0.02 (5.71e-01) | -0.08 (1.35e-01) |
| rs16891982 | **0.34 (6.45e-07)** | -0.13 (7.66e-02) | **-0.27 (5.06e-03)** |
| rs1325127 | **0.11 (7.37e-03)** | -0.03 (5.03e-01) | **-0.15 (1.06e-02)** |

Table S5. Betas and P-values from model 2. Significant contributions have been marked in bold.

| **Beta (Pval)** | **Nonpigm** | **Pheomel.** | **Eumel.** |
| --- | --- | --- | --- |
| mean | **-7.21 (5.03e-07)** | **4.46 (3.06e-03)** | 3.33 (1.11e-01) |
| rs1800407 | **-0.20 (8.79e-03)** | 0.03 (6.74e-01) | **0.44 (6.97e-05)** |
| rs2070959 | -0.06 (1.33e-01) | 0.01 (8.18e-01) | 0.07 (2.20e-01) |
| rs9894429 | 0.05 (1.52e-01) | -0.00 (9.21e-01) | -0.02 (7.56e-01) |
| rs1129038 | 0.47 (9.92e-02) | 0.16 (5.95e-01) | **-1.17 (4.83e-03)** |
| rs12203592 | **0.68 (4.88e-07)** | **-0.74 (2.59e-07)** | -0.19 (3.45e-01) |
| rs1393350 | **0.23 (8.74e-08)** | **-0.18 (6.22e-05)** | **-0.14 (2.27e-02)** |
| rs12913832 | **2.25 (4.71e-08)** | **-2.57 (3.22e-09)** | -0.81 (1.76e-01) |
| rs12896399 | 0.44 (1.61e-01) | -0.26 (4.41e-01) | -0.74 (1.11e-01) |
| rs3768056 | 0.05 (2.33e-01) | -0.03 (5.15e-01) | -0.10 (1.15e-01) |
| rs2835630 | 0.06 (1.12e-01) | -0.03 (4.36e-01) | -0.08 (1.27e-01) |
| rs16891982 | **1.64 (1.64e-09)** | **-1.33 (3.34e-06)** | **-0.81 (4.00e-02)** |
| rs1325127 | **0.43 (1.95e-05)** | **-0.39 (1.79e-04)** | **-0.26 (7.71e-02)** |
| rs12203592 x rs12913832 | **-0.26 (6.53e-04)** | **0.39 (9.56e-07)** | -0.08 (4.92e-01) |
| rs12896399 x rs16891982 | 0.14 (1.59e-01) | -0.21 (4.59e-02) | 0.01 (9.19e-01) |
| rs12913832 x rs12896399 | **-0.31 (1.96e-09)** | **0.32 (5.72e-09)** | **0.25 (7.48e-04)** |
| rs12913832 x rs1325127 | **-0.18 (4.84e-04)** | **0.20 (1.49e-04)** | 0.06 (3.97e-01) |
| rs12913832 x rs16891982 | **-0.72 (4.97e-15)** | **0.73 (6.12e-14)** | **0.24 (6.84e-02)** |

Table S6 Mean coefficient of determination (R^2 in ) from cross validation of different quantitative eye colour measurements using 6 IrisPlex SNPs.

| **Quantitative eye colour phenotype measure** | **Prediction Model 1 Without SNP-SNP interaction** | **Prediction Model 2 With SNP-SNP interaction** |
| --- | --- | --- |
| **No-pigmentation** | 52.47 (49.66, 55.78) | 53.05 (49.89, 56.32) |
| **Pheomelanin** | 16.27 (13.06, 19.70) | 17.64 (14.96, 20.19) |
| **Eumelanin** | 32.12 (28.99, 35.82) | 32.57 (29.69, 35.86) |
| **Hue** | 23.98 (20.89, 26.97) | 24.44 (21.49, 26.86) |
| **Saturation** | 42.29 (37.87, 46.37) | 42.94 (38.32, 47.41) |
| **Colour score** | 21.35 (16.99, 26.25) | 21.73 (16.89, 26.41) |
| **L** | 16.89 (13.54,20.27) | 17.19 (14.33,20.45) |
| **a*** | 40.98 (37.99,44.50) | 41.79 (38.72,44.92) |
| **b*** | 29.15 (25.55,32.52) | 30.02 (26.60,33.32) |
| **PIE-score** | 45.24 (41.79,47.70) | 46.58 (43.36,49.76) |
| **T-index** | 30.80 (27.30,34.56) | 32.09 (28.65,35.46) |

See Table 4 in the paper for further details.

Table S7. Betas and P-values from model 2 for SNPs as available on IrisPlex

| **Beta (Pval)** | **Nonpigm** | **Pheomel.** | **Eumel.** |
| --- | --- | --- | --- |
| mean | 0.32 (6.23e-01) | -0.40 (5.52e-01) | -3.74 (5.89e-05) |
| rs12913832 | -0.66 (2.23e-06) | -0.30 (4.15e-02) | 1.31 (9.55e-11) |
| rs1800407 | -0.17 (2.71e-02) | 0.01 (9.09e-01) | 0.41 (1.80e-04) |
| rs12896399 | 0.36 (2.58e-01) | -0.18 (6.02e-01) | -0.70 (1.29e-01) |
| rs16891982 | 0.03 (8.82e-01) | 0.29 (1.22e-01) | -0.26 (3.24e-01) |
| rs1393350 | 0.22 (3.74e-07) | -0.17 (1.51e-04) | -0.14 (2.75e-02) |
| rs12203592 | 0.75 (4.91e-08) | -0.80 (3.46e-08) | -0.21 (3.01e-01) |
| rs12203592 x rs12913832 | -0.29 (1.45e-04) | 0.42 (1.62e-07) | -0.07 (5.31e-01) |
| rs12896399 x rs16891982 | 0.18 (7.30e-02) | -0.25 (1.81e-02) | -0.00 (9.92e-01) |
| rs12913832 x rs12896399 | -0.32 (5.86e-10) | 0.33 (1.51e-09) | 0.26 (6.09e-04) |
| rs12913832 x rs16891982 | 0.02 (2.70e-01) | 0.02 (3.26e-01) | -0.06 (3.62e-02) |
